# How to make a more optimal surgical plan for Lenke 5 adolescent idiopathic scoliosis patients: a comparative study based on the changes of the sagittal alignment and selection of the lowest instrumented vertebra

**DOI:** 10.1186/s13018-023-03680-1

**Published:** 2023-03-21

**Authors:** Junyu Li, Zhengting Lin, Yinghong Ma, Weishi Li, Miao Yu

**Affiliations:** 1 Orthopaedic Department, Peking University Third Hospital, No. 49 North Garden Road, Haidian District, 100191 Beijing, China; 2 Engineering Research Center of Bone and Joint Precision Medicine, No. 49 North Garden Road, Haidian District, 100191 Beijing, China; 3 Beijing Key Laboratory of Spinal Disease Research, No. 49 North Garden Road, Haidian District, 100191 Beijing, China; 4 Peking University Health Science Center, No. 38 Xueyuan Road, Haidian District, 100191 Beijing, China

**Keywords:** Adolescent idiopathic scoliosis, Sagittal parameters, Lowest instrumented vertebra, Pelvic morphology

## Abstract

**Background:**

The treatment of patients with Lenke 5 adolescent idiopathic scoliosis (AIS) is closely related to the pelvic because the spine–pelvis is an interacting whole. Besides, the choice of fusion segment is a significant issue; with the optimal choice, there will be fewer complications and restoring the pelvic morphology to some extent. This study aims to analyze the impact of changes in sagittal parameters and selection of the lowest instrumented vertebra (LIV) on spine and pelvic morphology for better surgical strategy.

**Method:**

Ninety-four patients with Lenke 5 AIS who underwent selective posterior thoracolumbar/lumbar (TL/L) curve fusion were included in the study and grouped according to pelvic morphology and position of LIV. Spinopelvic parameters were measured preoperatively, postoperatively, and at the latest follow-up. The patient’s preoperative and last follow-up quality of life was assessed with the MOS item short-form health survey (SF-36) and scoliosis research society 22-item (SRS-22).

**Result:**

Patients being posterior pelvic tilt had the oldest mean age (*P* = 0.010), the smallest lumbar lordosis (LL) (*P* = 0.036), the smallest thoracic kyphosis (TK) (*P* = 0.399) as well as the smallest proximal junctional angle (PJA) while those being anterior pelvic tilt had the largest PJA. The follow-up TK significantly increased in both groups of anterior and normal pelvic tilt (*P* < 0.039, *P* < 0.006) while no significant changes were observed in the posterior pelvic tilt group. When LIV is above L4, the follow-up PJA was larger than other groups (*P* = 0.049, *P* = 0.006). When LIV is below L4, the follow-up TK and PT were larger and LL was smaller than other groups(*P* < 0.05). The SF-36 and SRS-22 scores were better in the LIV = L4 group than in other groups at the last follow-up (*P* < 0.05).

**Conclusion:**

The correction of TK and LL after surgery can improve pelvic morphology. Besides, LIV is best set at L4, which will facilitate the recovery of TK, the improvement of symptoms, and the prevention of complications and pelvic deformities.

**Level of evidence** Level III.

## Introduction

Adolescent idiopathic scoliosis (AIS) is a complex three-dimensional spinal deformity, which occurs in about 1.5%-3% of the young population and is manifested by the rotation and lateral bending of the vertebral body that result in abnormal coronal and sagittal alignment [1–3]. For patients with scoliosis greater than 40°, orthopedic surgery is usually required to prevent further progression of the deformity and restore the spine morphology [4].

At present, making the most optimal surgical plan has always been a major concern for spine surgeons, especially for Lenke type 5 patients, whose scoliosis is mainly located at the thoracolumbar or lumbar segment. Orthopedic surgery for them is more about fixation of the lumbar spine in the hope of reducing fixation to obtain more mobility, but too few fixed segments such as the lowest instrumented vertebra (LIV) above L3 can have many problems, such as trunk imbalance [5–7]. Besides, proportioned sagittal plane, such as postoperative pelvic morphology, is extremely important in terms of esthetics and quality of life, and complications such as proximal junctional kyphosis (PJK) and adding-on phenomenon may occur if poorly treated [2, 3, 8]. However, existing studies mostly focus on the correction of the coronal position of scoliosis patients, with much less attention paid to the postoperative changes in the sagittal plane and pelvic shape [2, 9]. Moreover, the influence of LIV on pelvic morphology is of great importance for the fact that the spine–pelvis is an interacting whole, so it is necessary to study the sagittal orthopedic parameters and the choice of the LIV [6, 9, 10].

This retrospective study aims to address such a gap in the literature by analyzing the effects of the changes in sagittal morphology and the selection of the lowest instrumented vertebra on the orthopedic outcomes and pelvic morphology. And the results will help inform the formulation of surgical plans for Lenke type 5 patients.

## Materials and methods

### Patients

Lenke 5 AIS patients who received posterior selective thoracolumbar or lumbar (TL/L) fusion and instrumented spinal fusion with pedicle screw fixation in our hospital from January 2007 to December 2018 with a minimum of two-year follow-up were retrospectively reviewed. Inclusion criteria were patients who 1) were diagnosed with Lenke type 5 AIS, 2) had undergone posterior internal fixation and fusion surgery to correct scoliosis, and 3) were followed up for at least two years. Exclusion criteria were patients with 1) insufficient follow-up time, 2) incomplete anteroposterior or lateral radiographs to obtain accurate spinal parameters, or 3) previous surgical treatments for other spinal abnormities. The subjects were grouped in two ways for comparative purposes. The first grouping method was based on the position of the pelvis at the last follow-up according to the approach proposed by Roussouly et al. [3, 11]. The patients’ pelvic tilt (PT) was less than (0.2 × PI / 2) in the anterior pelvic tilt group (AG), between (0.2 × PI / 2) and (0.8 × PI / 2) in the normal pelvis group (NG), and greater than (0.8 × PI / 2) in the posterior pelvic tilt group (PG). The second method was to divide patients into three groups according to the position of the lowest instrumented vertebra (LIV): L3 or above, L4, and L5 or below [12, 13]. This study was approved by the institutional review board of our hospital.

### Measurements and parameters

**Fig. 1:**
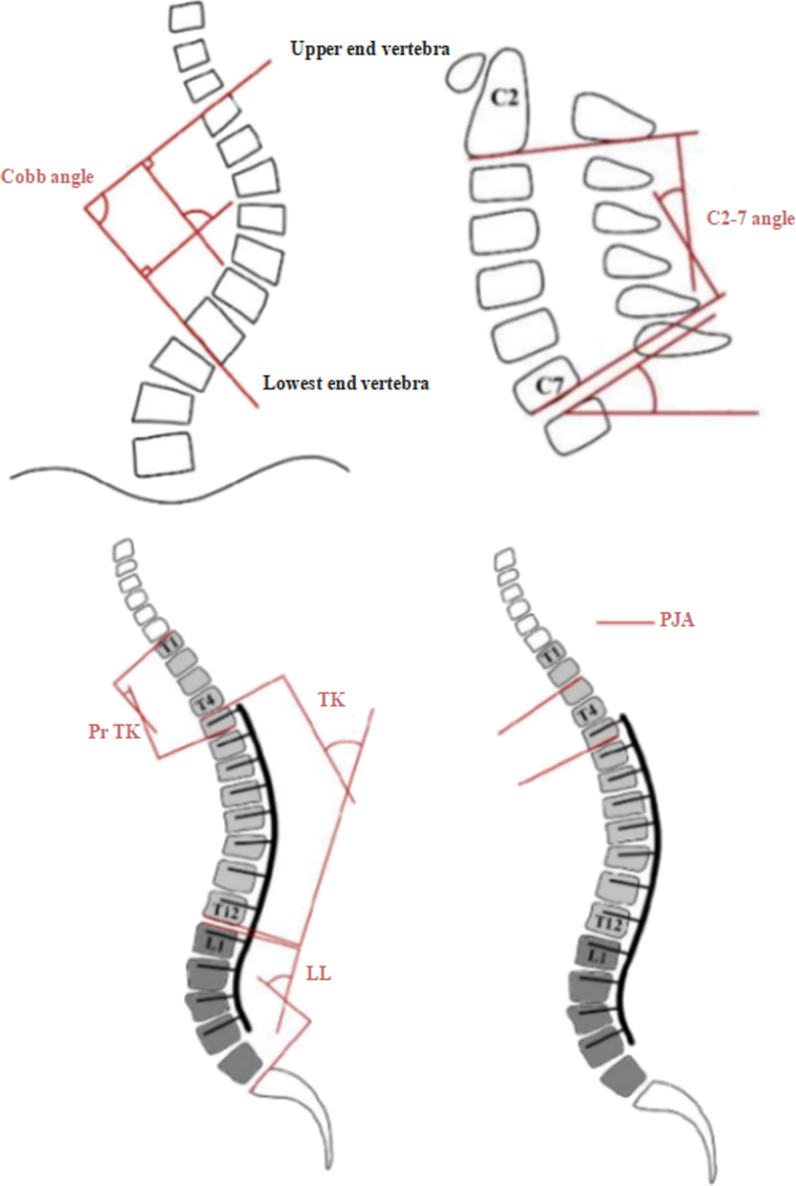
Measurement of coronal parameters (including Cobb angle), sagittal parameters (including CL and T1 slope), descriptive parameters (including PrTK, TK, and LL), and the measurement of PJA [proximal junctional angle, the angle between the inferior endplate of the upper instrumented vertebra (UIV) and the superior endplate of the UIV + 2]

We examined the subjects’ preoperative, postoperative, and final follow-up anteroposterior and lateral radiographs of the entire spine and determined their Lenke type based on the X-ray bending radiographs. Patients’ age was obtained from their medical records. Radiological parameters were measured as follows: 1) major thoracolumbar/lumbar (TL/L) curve Cobb angle; 2) cervical lordosis (CL, C2–7 Cobb angle); 3) T1 slope; 4) thoracic kyphosis (TK, T4-12); 5) proximal thoracic kyphosis (PrTK, T1-T5); 6) lumbar lordosis (LL, L1–S1); 7) proximal junctional angle (PJA) (Fig. 1); 8) pelvic incidence (PI); 9) pelvic tilt (PT); and 10) sacral slope (SS). Proximal junctional kyphosis (PJK) is a common complication in AIS patients with a major thoracic curve which is diagnosed when the PJA met the following two criteria: 1, PJA ≥ 10°; and 2, at least 10° greater than the preoperative measurement [14]. The classification was conducted by five researchers, who measured the above spinal parameters and determined the patient’s specific type.

### Statistical analyses

Statistical analysis was performed with SPSS version 22.0. The value of all the parameters was presented in the form of mean ± standard deviation (SD). Single-factor analysis of variance was used to examine intergroup differences. Differences between preoperative and follow-up values of the parameters for the same patient were analyzed with paired sample t test. For data with a small sample size or non-normal distribution, intergroup differences were examined with sign test. Chi-square test or Fisher’s exact test was used to analyze the influence of LIV selection on parameter changes. Statistical significance was set at *P* < 0.05.

## Results

A total of 94 AIS patients, of which the sex ratio was 78 females to 16 males, were enrolled in this study (Table 1). The patients’ mean age at operation was 15.4 ± 2.8 years old (range: 12–22), the mean follow-up time was 32.5 ± 12.6 (24–82) months, and the mean preoperative Cobb angle was 45.0° ± 13.7° (43°–73°).

**Table 1:** Demographic and clinical features of all cases

Case	*N* = 94
Age at surgery (year)	15.4 ± 2.8(12–22)
Sex (female/male)	78/16
FU (months)	32.5 ± 12.6(24–82)
Preoperative major TL/L curve(°)	45.0 ± 13.7(43− 73)

Values indicate mean ± standard deviation

*year* indicates years; *FU* follow-up period; *M* minor; *N* normal; *H* high; *TL/L* thoracolumbar/lumbar

Table 2 shows the differences among preoperative, postoperative, and follow-up parameters of all subjects. The Cobb angle decreased from 45.0° ± 13.7° before surgery to 15.6° ± 10.2° at the follow-up (*P* < 0.001), and TK increased from 22.5° ± 15.6° preoperatively to 28.2° ± 16.7° at the follow-up (*P* = 0.003).

**Table 2:** Comparison of perioperative and follow-up parameters in all cases

	Preoperative	Postoperative	*P*-pre and post	Follow-up	*P*-pre and follow-up	*P*-post and follow-up
Major TL/L curve (°)	45.0 ± 13.7	14.1 ± 10.5	< 0.001*	15.6 ± 10.2	< 0.001*	0.190
CL (°)	4.0 ± 12.3	3.9 ± 10.2	0.607	0.9 ± 15.4	0.143	0.043*
PrTK (°)	7.4 ± 7.1	11.0 ± 7.7	0.001*	8.0 ± 8.7	0.890	0.031*
TK (°)	22.5 ± 15.6	24.8 ± 10.9	0.199	28.2 ± 16.7	0.003*	0.160
LL (°)	− 51.4 ± 12.2	− 50.6 ± 12.5	0.621	− 51.3 ± 20.9	0.212	0.991
PI (°)	48.3 ± 12.4	46.8 ± 15.2	0.246	45.9 ± 19.4	0.871	0.517
PT (°)	9.2 ± 7.7	7.9 ± 9.5	0.239	9.5 ± 11.2	0.887	0.341
SS (°)	39.2 ± 8.5	43.1 ± 27.4	0.307	36.6 ± 14.7	0.532	0.108
PJA (°)	7.6 ± 5.6	6.6 ± 9.5	0.391	8.1 ± 10.1	0.486	0.045*
T1 slope (°)	14.0 ± 8.7	17.1 ± 8.7	0.138	16.1 ± 9.7	0.252	0.986

Values indicate mean ± standard deviation

*Significant difference

*PI* indicates pelvic incidence; *PT* pelvic tilt; *SS* sacral slope; *LL* lumbar lordosis; *PrTK* proximal thoracic kyphosis; *TK* thoracic kyphosis; *CL* cervical lordosis; *pre* preoperative; *post* postoperative; *TL/L* thoracolumbar/lumbar

### Comparison within and between pelvic tilt groups

The differences within and between the three pelvic tilt groups (AG, NG, and PG groups) are shown in Table 3. In all three groups, Cobb angle was significantly improved after surgery as well as at the last follow-up (*P* < 0.05).

**Table 3:** Comparison of differences between pelvic position groups

Age (year)	AG (*N* = 28)				NG (*N* = 42)				PG (*N* = 24)				*P* value among the three groups		
	15.6 ± 3.2				14.9 ± 2.7				21.0 ± 1.0				0.010*		
	Pre-op	Post-op	L-FU	*P*-value	Pre-op	Post-op	L-FU	*P*-value	Pre-op	Post-op	L-FU	*P*-value	Pre-op	Post-op	L-FU
Major TL/L curve(°)	40.7 ± 10.2	15.1 ± 12.0	11.3 ± 6.5	< 0.001*	41.6 ± 11.3	13.9 ± 9.8	17.3 ± 11.7	< 0.001*	46.6 ± 13.3	9.7 ± 5.9	15.7 ± 3.4	0.013*	0.672	0.677	0.351
CL (°)	0.1 ± 14.2	3.3 ± 12.2	4.4 ± 16.9	0.665	5.3 ± 10.6	5.3 ± 8.5	3.0 ± 10.8	0.075	0.3 ± 7.7	2.7 ± 4.7	6.8 ± 11.5	0.893	0.527	0.848	0.38
PrTK (°)	10.0 ± 7.1	10.0 ± 8.3	7.1 ± 8.7	0.412	7.3 ± 5.7	12.3 ± 7.4	7.5 ± 7.2	0.843	13.9 ± 3.7	15.4 ± 5.7	8.8 ± 3.2	0.625	0.155	0.493	0.936
TK (°)	30.2 ± 13.3	30.2 ± 12.3	32.2 ± 27.6	0.039*	21.9 ± 11.7	25.9 ± 9.5	29.2 ± 11.1	0.006*	15.9 ± 13.7	17.6 ± 16.6	16.9 ± 15.5	0.871	0.14	0.213	0.399
LL (°)	− 53.7 ± 12.5	− 56.6 ± 10.2	− 57.1 ± 36.5	0.778	− 51.1 ± 15.8	− 52.1 ± 13.4	− 53.2 ± 14.3	0.981	− 48.3 ± 6.9	− 39.9 ± 6.1	− 36.3 ± 6.8	0.035*	0.797	0.077	0.036*
PI (°)	46.3 ± 11.8	47.7 ± 16.2	44.5 ± 19.7	0.27	49.8 ± 14.6	47.7 ± 17.7	46.8 ± 12.8	0.272	55.7 ± 13.4	54.7 ± 15.8	58.1 ± 16.6	0.625	0.516	0.812	0.152
PT (°)	5.2 ± 4.2	3.9 ± 6.4	1.4 ± 9.6	0.054	12.3 ± 8.4	9.0 ± 9.8	10.7 ± 6.1	0.411	10.4 ± 10.1	19.6 ± 17.5	28.2 ± 6.6	0.125	0.107	0.055	< 0.001*
SS (°)	41.0 ± 10.9	43.9 ± 11.1	45.9 ± 24.0	0.555	37.5 ± 10.0	38.8 ± 11.6	36.1 ± 9.4	0.474	45.4 ± 7.5	34.1 ± 6.7	29.9 ± 10.1	0.625	0.36	0.302	0.048*
PJA (°)	7.9 ± 4.6	6.7 ± 4.4	9.2 ± 4.5	0.094	7.5 ± 5.8	3.7 ± 8.2	6.6 ± 11.2	0.037*	9.5 ± 7.0	2.8 ± 8.7	3.4 ± 6.6	0.625	0.835	0.539	0.575
T1 slope (°)	20.9 ± 11.3	21.2 ± 10.6	22.3 ± 7.7	0.91	12.0 ± 6.0	15.6 ± 7.1	15.3 ± 8.2	0.152	13.3 ± 5.5	20.5 ± 3.8	16.9 ± 3.3	0.794	0.056	0.283	0.115

Values indicate mean ± standard deviation

*Significant difference

*AG* anterior pelvic tilt group; *NG* normal pelvis group; *PG* posterior pelvic tilt group; *Pre-op* preoperative; *Post-op* postoperative; *L-FU* last follow-up; *PI* pelvic incidence; *PT* pelvic tilt; *SS* sacral slope; *LL* lumbar lordosis; *PrTK* proximal thoracic kyphosis; *TK* thoracic kyphosis; *CL* cervical lordosis; *TL/L* thoracolumbar/lumbar

In AG, TK increased from 30.2 ± 13.3 preoperatively to 32.2 ± 27.6 at the follow-up (*P* = 0.039). In the NG group, TK increased from 21.9 ± 11.7 preoperatively to 29.2 ± 11.1 at the follow-up (*P* = 0.006). There is no significant difference in terms of TK in the PG group and LL in each group during different periods. Among the three groups, PG had the oldest mean age (*P* = 0.010), the smallest LL(*P* = 0.036), the smallest TK(*P* = 0.399) as well as the smallest PJA(*P* = 0.575) while AG had the largest PJA.

### Comparison within and between LIV groups

The differences within and between the three LIV groups (LIV ≥ L3, LIV = L4, and LIV ≤ L5 groups) are shown in Table 4. In all three groups, Cobb angle was significantly improved both after surgery and at the follow-up (*P* < 0.05). In the LIV ≥ L3 group, the PJA increased from 8.8 ± 4.7 preoperatively to 9.7 ± 6.0 at the follow-up (*P* = 0.011). In the LIV = L4 group, TK increased from 22.6 ± 12.7 preoperatively to 33.1 ± 13.3 at the follow-up (*P* < 0.001), and PJA increased from 6.9 ± 5.2 preoperatively to 8.5 ± 5.2 at the follow-up (*P* = 0.044). In the LIV ≤ L5 group, Cobb angle decreased from 50.0 ± 12.2 preoperatively to 20.2 ± 16.7 at the follow-up (*P* = 0.033). There were significant differences in both postoperative and follow-up PT values among the three groups. In the LIV ≥ L3, LIV = L4, and LIV ≤ L5 groups, the postoperative PT was, respectively, 10.5 ± 10.7, 5.1 ± 7.4, and 21.1 ± 15.9 (*P* = 0.020), and the follow-up PT was, respectively, 5.1 ± 14.2, 7.3 ± 9.4, and 24.0 ± 11.1 (*P* = 0.023). Besides, the postoperative and follow-up PJA values in the LIV ≥ L3 group were the largest among the three groups(*P* < 0.05). The LL was, respectively, − 54.2 ± 11.0, − 54.8 ± 13.1, and − 40.2 ± 14.6 at the last follow-up (*P* = 0.047). The SF-36 and SRS-22 scores were better in the LIV = L4 group than in other groups at the last follow-up (*P* = 0.037).

**Table 4:** Comparison of differences in LIV grouping parameters

Age (year)	≥ L3 group (*N* = 22)				L4 group (*N* = 41)				≤ L5 group (*N* = 31)				*P* value among the three groups		
	14.5 ± 2.8				16.0 ± 3.4				18.0 ± 3.5				0.155		
	Pre-op	Post-op	L-FU	*P*-value	Pre-op	Post-op	L-FU	*P*-value	Pre-op	Post-op	L-FU	*P*-value	Pre-op	Post-op	L-FU
Major TL/L curve(°)	38.2 ± 13.6	14.4 ± 10.3	15.7 ± 10.7	0.034*	42.8 ± 9.7	12.6 ± 8.8	13.0 ± 7.0	< 0.001*	50.0 ± 12.2	15.4 ± 15.4	20.2 ± 16.7	0.033*	0.255	0.847	0.394
CL (°)	7.1 ± 9.7	8.4 ± 10.1	4.8 ± 16.4	0.565	3.1 ± 13.5	1.9 ± 8.8	2.9 ± 13.1	0.113	1.1 ± 4.5	4.2 ± 10.1	7.5 ± 10.8	0.304	0.535	0.315	0.264
PrTK (°)	7.5 ± 6.1	10.1 ± 4.7	5.2 ± 9.6	0.21	9.1 ± 6.5	11.5 ± 8.5	8.0 ± 6.8	0.612	12.0 ± 3.9	17.0 ± 3.2	8.5 ± 3.3	0.193	0.524	0.314	0.655
TK (°)	29.5 ± 14.5	29.6 ± 6.3	22.1 ± 27.5	0.618	22.6 ± 12.7	25.9 ± 12.7	33.1 ± 13.3	< 0.001*	16.7 ± 8.1	19.9 ± 14.3	17.1 ± 14.9	0.95	0.27	0.424	0.189
LL (°)	− 56.1 ± 11.8	− 56.9 ± 13.7	− 54.2 ± 11.0	0.534	− 51.9 ± 13.9	− 51.3 ± 11.8	− 54.8 ± 13.1	0.418	− 51.0 ± 8.7	− 45.2 ± 10.3	− 40.2 ± 14.6	0.881	0.189	0.313	0.047*
PI (°)	48.7 ± 17.0	51.7 ± 21.7	49.8 ± 20.8	0.688	48.4 ± 11.7	45.0 ± 13.9	47.1 ± 9.0	0.143	54.7 ± 14.7	57.9 ± 12.8	58.4 ± 17.2	0.49	0.704	0.305	0.267
PT (°)	9.3 ± 7.4	10.5 ± 10.7	5.1 ± 14.2	0.313	7.9 ± 7.7	5.1 ± 7.4	7.3 ± 9.4	0.825	17.5 ± 5.4	21.1 ± 15.9	24.0 ± 11.1	0.287	0.082	0.020*	0.023*
SS (°)	39.4 ± 10.5	41.2 ± 13.3	43.2 ± 27.4	0.453	40.5 ± 10.0	40.0 ± 11.1	36.8 ± 10.0	0.248	37.2 ± 10.2	36.9 ± 5.5	34.3 ± 7.4	0.472	0.834	0.824	0.864
PJA (°)	8.8 ± 4.7	7.0 ± 5.9	9.7 ± 6.0	0.011*	6.9 ± 5.2	6.2 ± 4.2	8.5 ± 5.2	0.044*	12.4 ± 6.5	5.9 ± 10.8	5.5 ± 15.2	0.895	0.182	0.003*	0.006*
T1 slope (°)	15.7 ± 10.5	14.3 ± 7.1	17.0 ± 10.8	0.647	13.9 ± 9.3	18.3 ± 8.9	17.5 ± 9.0	0.238	14.0 ± 6.9	22.3 ± 6.9	15.9 ± 1.2	0.587	0.913	0.353	0.946
SF-36	43.2 ± 4.1	–	39.7 ± 4.5	0.039*	44.5 ± 5.1	–	41.7 ± 4.3	0.043*	43.5 ± 5.4	–	33.7 ± 5.5	0.027*	0.718	–	0.037*
SRS-22	4.6 ± 0.8	–	4.0 ± 0.1	0.044*	4.5 ± 0.4	–	4.2 ± 0.5	0.037*	4.4 ± 0.3	–	3.7 ± 0.5	0.012*	0.634	–	0.037*

Values indicate mean ± standard deviation

*Significant difference

When LIV > L3, it means LIV is higher than L3. *Pre-op* preoperative; *Post-op* postoperative; *L-FU* last follow-up; *PI* pelvic incidence; *PT* pelvic tilt; *SS* sacral slope; *LL* lumbar lordosis; *PrTK* proximal thoracic kyphosis; *TK* thoracic kyphosis; *CL* cervical lordosis; *TL/L* thoracolumbar/lumbar

### Complications and revisions

Among the three groups, the overall number of complications was similar. The revision rate between groups LIV ≥ L3 and LIV ≤ L5 was similar (27.3% vs. 25.8%; *P* = 0.332). Furthermore, the revision rate of group LIV = L4 (19.5%) is considerably lower than that of the other two groups. In group LIV ≥ L3, the distal extension was the primary cause of revision (50.0%). Between the three groups, there was no difference in revisions as a result of adding-on (4.55% vs. 4.88% vs. 3.23%; *P* = 0.611). However, the adding-on manifested in five patients in group LIV ≥ L3, one of whom required a revision operation. There was 2 revision due to PJK in group LIV ≥ L3, but no discernible difference was seen between the three groups' total junctional failure (*P* = 0.367). In neither group were there any mortalities or repeated revisions. There were no mortalities or recurrent revisions in either group (Table 5). Through subgroup analyses, 6 patients with L5 LIV in group LIV ≤ L5 were identified. Of the 3 patients who needed revision, 1 patient needed revision due to distal extension, 1 patient needed revision due to PJK, and 1 patient needed revision due to adding-on.

**Table 5:** Complications and revisions in different groups

Variables	≥ L3 group (*N* = 22)	L4 group (*N* = 41)	≤ L5 group (*N* = 31)	*P*-value
Total number of complications	28	33	26	0.312
Major complication type	3	2	7	0.066
Implants	0(0%)	0(0%)	2(7.69%)	0.087
Neurologic	0(0%)	0(0%)	2(7.69%)	0.087
Cardiopulmonary	1(3.57%)	0(0%)	1(3.85%)	0.524
Infection	2(7.14%)	1(6.06%)	0(0%)	0.504
Operative	0(0%)	1(6.06%)	2(7.69%)	0.387
Gastrointestinal	0(0%)	0(0%)	0(0%)	–
Renal	0(0%)	0(0%)	0(0%)	–
Vascular	0(0%)	0(0%)	0(0%)	–
Mortality	0(0%)	0(0%)	0(0%)	–
Total patient affected (%)	4(18.2%)	5(12.2%)	6(19.4%)	0.716
Total number of revisions	6(27.3%)	8(19.5%)	8(25.8%)	0.704
Recurrent revision	0	0	0	–
Revision type				0.400
Proximal junctional kyphosis	2(9.09%)	1(2.44%)	1(3.23%)	
Adding-on	1(4.55%)	2(4.88%)	1(3.23%)	
Implants failure	0(0%)	0(0%)	3(9.68%)	
Distal extension	3(13.6%)	2(4.88%)	1(3.23%)	
Others	0(0%)	3(7.32%)	2(6.45%)	

When LIV > L3, it means LIV is higher than L3. When LIV < L5, it means LIV is lower than L5

## Discussion

At present, making the most optimal surgical plan has always been a major concern for spine surgeons, especially for Lenke type 5 patients. However, studies on the effects of posterior orthopedic surgeries on Lenke type 5 patients mostly focus on the correction of the coronal plane, with less attention paid to the postoperative changes in the sagittal alignment and pelvic shape. Besides, the selection of the LIV, which remains a controversial issue, is also important due to the interaction impact between the spine and pelvis; with the optimal choice, we can reduce complications and improve the pelvic morphology to some extent. In light of such gaps, this study focused on Lenke type 5 AIS patients and explored the impact of the change in sagittal parameters and the selection of different intraoperative fusion segments on the orthopedic effect and pelvic morphology.

The Cobb angle of all 94 patients in this study was significantly improved after surgery and at the follow-up compared with that before surgery. The correction rate immediately after surgery was 68% and that at the end of the two-year follow-up was 65%, which implied success in surgical correction and was consistent with the report of Abel et al. [15]. In addition, TK somewhat recovered at the last follow-up, which suggested that posterior orthopedic surgery could correct the thoracic curvature and was consistent with the report by Chen et al. [14].

### Postoperative changes in pelvic morphology

The orthopedic range of Lenke type 5 AIS patients is located at the TL/L segment, which is adjacent to the pelvis, and the lumbar curvature is also impacted by the pelvis to a certain extent [2, 8, 16, 17]. Pelvic morphology is an important consideration for the evaluation of sagittal balance in terms of esthetics and quality of life, and complications such as proximal junctional kyphosis (PJK) and adding-on phenomenon may occur if poorly treated [13, 18, 19]. Therefore, it is worth further studying whether posterior orthopedic surgeries will affect the pelvic morphology among these patients.

According to the approach proposed by Roussouly et al. [11], we divided the pelvic morphology into three categories based on PT at the last follow-up: anterior pelvic tilt, normal pelvic tilt, and posterior pelvic tilt. Xu et al. [2] observed that 25% of Lenke type 5 patients had anterior pelvic tilt and concluded that the increase of LL and SS was significantly associated with the risk of unsuccessful postoperative recovery from forward pelvic tilt, which was consistent with our results. In our study, only 6% of patients had a forward pelvic tilt and 8% had a backward pelvic tilt before surgery. As the patient reflexively rebalances to keep his head above his feet, overcorrection and severe anterior convexity of the lumbar spine may result in PJK, since it causes the adjacent vertebrae above the UIV to tilt forward. In our research, LL in AG was larger than that in NG at the final follow-up, suggesting that LL was a risk factor for abnormal anterior tilt pelvic shape. What is more, among the three groups, the LL value of patients in PG was the smallest (*P* = 0.036) and tended to decrease without enough correction, which indicates that the decrease of LL is also a serious risk factor for surgery-related abnormal posterior tilt pelvic shape. Besides, most of the existing studies maintained that the change of TK was a compensatory reaction to the change of LL [2, 8, 16]. The follow-up TK significantly increased in both AG and NG, but decreased and was the smallest in the PG, which along with the decrease of LL implied the risk of flat back deformity. It was also clear that PJA in AG had a tendency to increase, which indicates the possibility of PJK and PJF in the future. Therefore, the repair of TK and LL must be given priority throughout the surgery since the lumbar spine is the key surgical segment in patients with Lenke 5 AIS.

Besides, our results indicated that younger patients tended to have a forward pelvic tilt while older patients tended to have a backward pelvic tilt (*P* = 0.010). Therefore, older age was considered an independent risk factor for a backward pelvic tilt, which may be related to age-induced spinal degeneration [20–22].

In summary, the correction of their TK and LL is critical during the operation to improve the postoperative pelvic shape. Besides, age affects pelvic tilt and should be considered when planning surgery.

### Effect of LIV on surgical outcome

**Fig. 2:**
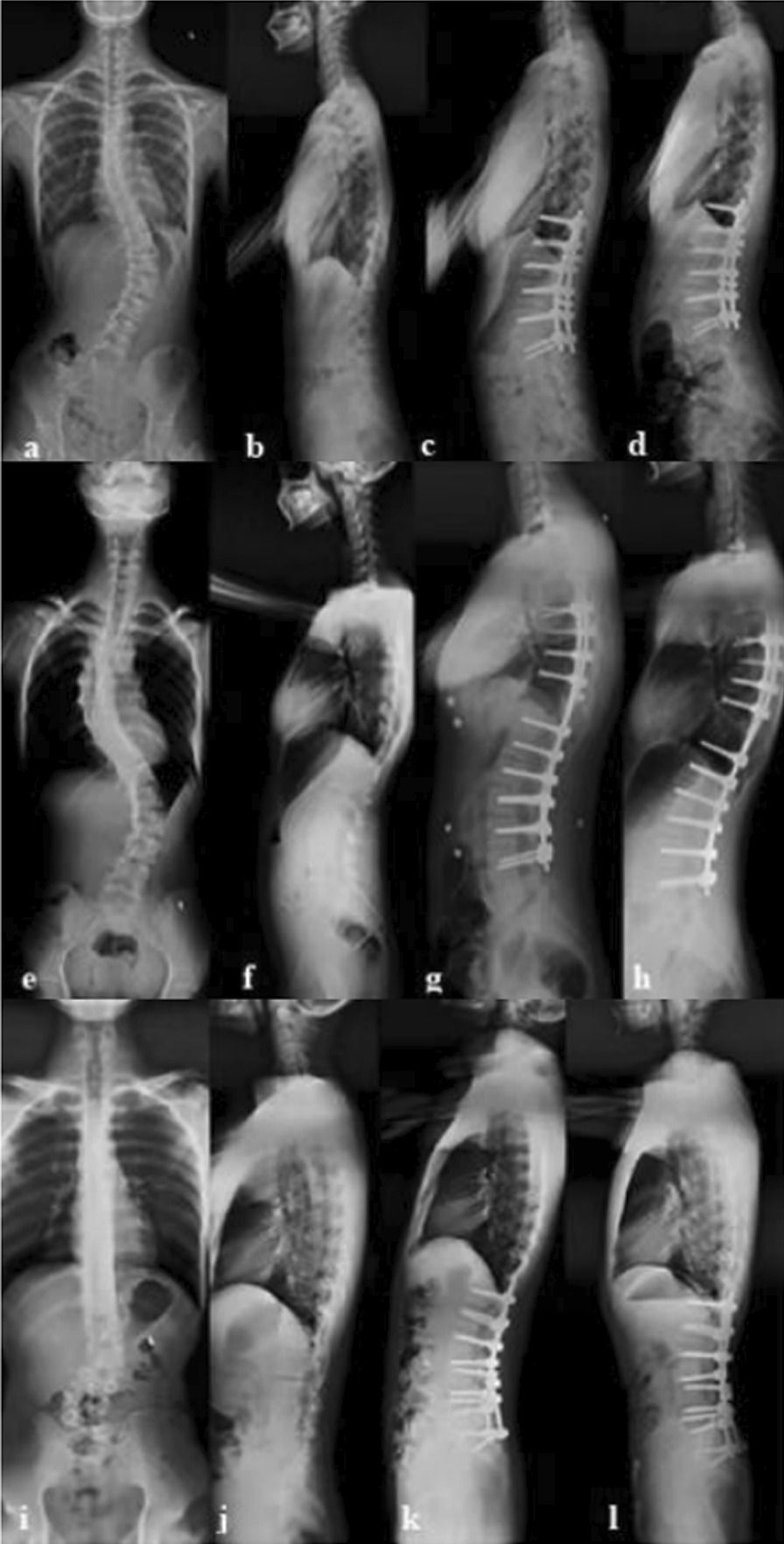
Effect of different LIV on sagittal alignment. In the pictures, 1) the patient whose LIV was at L3 was shown in a, b, c, and d. PT, LL, TK, and PJA in the preoperative period were 11.9°, − 40.9°, 31.8°, and 1.6° (**a**, **b**). PT, LL, TK, and PJA in the postoperative period were 1.4°, − 31.9°, 25.1°, and 0.3° (**c**). PT, LL, TK, and PJA in the latest follow-up were 10.3°, − 76.6°, 54.7°, 29.9° (**d**); 2) patient whose LIV was at L4 was shown in e, f, g, and h. PT, LL, TK, and PJA in the preoperative period were 10.2°, − 33.2°, 23.5°, and 4.5° (**e**, **f**). PT, LL, TK, and PJA in the postoperative period were 2.6°, − 44.7°, 41.2°, and 2.6° (**g**). PT, LL, TK, and PJA in the latest follow-up were11.2°, − 55.7°, 45.6°, and 2.4° (**h**). 3) Patient whose LIV was at the sacrum was shown in **i–l**. PT, LL, TK, and PJA in the preoperative period were 13.2°, − 3.4°, 24.4°, and 3.1° (**i**, **j**). PT, LL, TK, and PJA in the postoperative period were 15.9°, − 23.8°, 34.8°, and 1.9° (**k**). PT, LL, TK, and PJA in the latest follow-up were 2.4°, − 49.3°, 29.6°, and 13.9° (**l**)

Lenke type 5 patient alignment plans have been hotly contested, and the choice of surgical segments—particularly the LIV—also affects sagittal parameters [6, 14] (Fig. 2).

As for pelvic morphology, Yang et al. [3] have reported that choosing short segmental fusion is more conducive to retaining more lumbar mobility, thus preventing postoperative pelvic forward tilting, whose results are similar to ours. They also reported that the risk of trunk imbalance for patients with the LIV at L5 was very high [13]. Indeed, when LIV is higher than or equal to L4, the pelvis is more mobile and serves an important compensatory role for the trunk to effectively maintain trunk balance. Every attempt should be made to avoid sacrificing lumbar motion segments to obtain more coronal correction of scoliosis. We found when LIV was lower than L4, the pelvis tended to tilt backward; but when LIV was higher than or equal to L4, the pelvis tends to be normal, which is the result of the interaction between the spine and the pelvis. As LL was included in the posterior fusion, LL below the LIV would be subject to compensatory changes so that LL would be geometrically stable with the pelvis. A higher fusion segment indicates more compensated parts below the fusion segment [3, 23], and LL is relatively large, leading to reduced PT [2], and pelvic backward tilt is prone to recovery [13, 24]. PT is also an important parameter to evaluate sagittal plane balance, and a large PT (PT > 20°) is an indication of pelvis tilt backward and postoperative sagittal imbalance [13]. Additionally, the literature has shown reports of lack of correction over fused segments. Banno et al. [25] found that among 63 individuals with ASD who had lower thoracic fusion to the pelvis, there was a 27% loss of correction. Furthermore, similar to our case, they reported that loosening of the iliac screw suggested instability of the lumbosacral junction, which might lead to sagittal malalignment and a poor outcome [26]. Longer follow-up increased the chance of correction loss in lower fixed segments (LIV is below L4), but the difference was not statistically significant in our study. Fusions that terminate above L5 may preserve lumbosacral motion, lessen stress on the sacroiliac joint, require less extensive dissection, and reduce the risk of pseudoarthrosis [8, 25, 27]. The recovery of pelvic form and the maintenance of the corrected spinal–pelvic morphology has a greater impact on the quality of life of the patient. Therefore, a lower LIV which is below L4 is not recommended.

The risk of complications and revision are also essential factors to consider when determining the distal fusion level [7, 10, 14, 26]. The most common reasons for revision are adding-on phenomena, implant-related complications, PJK, infection, and adjacent segment degeneration [3, 13, 14]. The two main reasons for revision in patients with a LIV above L4 are symptomatic adjacent segment degeneration and the requirement for a sacral extension. Previous research has demonstrated that lower fixed vertebrae are at risk for PJK, primarily as a result of stress on a smaller TK and larger SVA [22, 28]. At the 2-year follow-up, we discovered that sagittal alignment restoration was preserved in the groups LIV = L4 and LIV ≤ L5, whereas a significant decrease of correction was discovered in the group LIV ≥ L3. Increased kyphosis at the unfused spine, such as junctional kyphosis or the reciprocal shift of TK, is linked to sagittal decompensation [28]. The comparatively large LL has a compensatory impact on the pelvis when LIV is greater than or equal to L3, resulting in an anterior pelvic tilt. The body will concurrently tend to tilt forward in the segment above the thoracolumbar to maintain the body's center of gravity. This causes the overall kyphotic correction of the spine to be relatively insufficient, leading to higher PJA. According to Wang et al. [29], postoperative PJA was a compensatory adjustment for sagittal spinal disorders and was linked to a higher incidence of PJK. In our study, compared to L4 and L5 groups, the revision rate for PJK was highest in group L3 (9.09%), but the difference was not statistically significant.

Besides, previous studies surfaced that higher vertebrae suffer from slippage and anterior–posterior sway led to an increased incidence of the adding-on phenomenon [27, 30], which is similar to our experimental results. Hua et al. [27] showed that postoperative LIV translation and postoperative coronal imbalance could be determined as risk factors for postoperative distal adding-on in patients with Lenke 5C AIS. Ohrt-Nissen et al. [30] found leaving unfused segments in the lower spine carries the risk of adding-on though increasing the potential for compensatory mechanisms to improve spinal and truncal balance. Our study showed that the incidence of adding-on phenomena was higher in patients in group LIV ≥ L3 than in the other two groups, although few required revision because of symptoms, and SRS-22 scores were not adversely affected in patients with distal add-on during the follow-up period. Since distal adding-on phenomena can have adverse effects on the lumbar spine, such as accelerating the degenerative process, longer follow-up of patients is needed to further elucidate this issue.

In addition, in patients with a LIV above L4, symptomatic adjacent segment degeneration and the need for an extension to the lower segment even sacrum are the major indications for revision. The reported revision rate for an extension to the sacrum is 23% [31]. In our study, there were more patients (13.6% vs.4.88%, 3.23%; *P* = 0.103) undergoing distal extension revision in group LIV ≥ L3 than in the other two groups. And in groups LIV ≥ L3 and LIV ≤ L5, total rates of revision are both higher than group LIV = L4 (27.3%, 25.8% vs.19.5%), which is similar to the research of Chen et al. [32] Our study also showed that when LIV was equal to L4, especially the recovery of TK, SF-36, and SRS-22 was the best. So a higher LIV which is above L4 is not recommended in terms of the risk of complications and needed revision.

In summary, our study suggested L4 was the optimal segment selected as LIV in spinal orthodontics, in which case risks of sagittal imbalance, complications, and revision can be minimized and the spine curvature will have the best recovery outcome in terms of TK, LL, and pelvic morphology.

There are some limits to this study. Firstly, the sample size is limited in a single-center study. Secondly, we did not take into account how patients might be affected by the choice of the upper instrumented vertebra. Therefore, a multicenter, prospective, randomized study with longer follow-up is needed to examine our present outcomes.

## Conclusion

For Lenke type 5 patients, surgical plans for posterior scoliosis correction must pay attention to the changes in sagittal statutes. Appropriate correction of TK and LL after surgery can improve pelvic morphology. In terms of the selection of fusion level, we suggest that LIV is best set at L4, which will facilitate the recovery of TK, the improvement of symptoms, and the prevention of PJK and pelvic deformities.

## Abbreviations

PI: Pelvic incidence
PT: Pelvic tilt
SS: Sacral slope
LL: Lumbar lordosis
PrTK: Proximal thoracic kyphosis
TK: Thoracic kyphosis
CL: Cervical lordosis
pre: Preoperative
post: Postoperative
L-FU: Last follow-up
TL/L: Thoracolumbar/lumbar
AG: Anterior pelvic tilt group
NG: Normal pelvis group
PG: Posterior pelvic tilt group
PJA: Proximal junctional angle
PJK: Proximal junctional kyphosis
LIV: The lowest instrumented vertebra
